# More why, less how: What we need from models of cognition^☆^

**DOI:** 10.1016/j.cognition.2021.104688

**Published:** 2021-08

**Authors:** Dennis Norris, Anne Cutler

**Affiliations:** a MRC Cognition and Brain Sciences Unit Cambridge, United Kingdom; b The MARCS Institute and Centre of Excellence for the Dynamics of Language, Western Sydney University, Australia

**Keywords:** Cognition, Theory, Word recognition

## Abstract

Science regularly experiences periods in which simply describing the world is prioritised over attempting to explain it. *Cognition*, this journal, came into being some 45 years ago as an attempt to lay one such period to rest; without doubt, it has helped create the current cognitive science climate in which theory is decidedly welcome. Here we summarise the reasons why a theoretical approach is imperative in our field, and call attention to some potentially counter-productive trends in which cognitive models are concerned too exclusively with how processes work at the expense of why the processes exist in the first place and thus what the goal of modelling them must be.

## Highlights

• We suggest that theories should try to explain ‘why’ not just ‘how’.
• We reinforce Darwin’s (1861) point that “All [scientific] observation must be for or against some view if it is to be of [scientific] service!”.
• Jacques Mehler and Cognition revitalised theory in cognitive psychology.

Some issues just won't go away. Consider this: “… there was [then] much talk that geologists ought only to observe and not theorise; and I well remember someone saying that at this rate a man might as well go into a gravel pit and count the pebbles and describe the colours. How odd it is that anyone should not see that all observation must be for or against some view if it is to be of any service!” The writer of this remark? Charles Darwin, in September of 1861 (Darwin, 1861)

Fast forward to a little over a century later. Experimental psychology had by then become an established field, and was in the process of experiencing a wave of change that was later deemed to have been revolutionary in nature, with the behaviorist era yielding to the Cognitive Revolution. To that date, language (except perhaps as represented by lists of unrelated words) had played little or no role in experimental psychology, which in the behaviorist time was firmly empiricist in its goals and in consequence austerely minimalist in its practice. Experimental psychology publications contained descriptive reports of experimental studies which were no more than that: reports of how an experiment was done and what its results were. At that time “Authors knew that to enhance their chances of publication they had to avoid motivating their studies theoretically” (Mehler, 1994). As language became more common as a topic of research, “there were barely two psychology journals where we and our colleagues could hope to publish: *The Journal of Verbal Learning and Verbal Behavior*, *Psychological Review*. Both were dominated by behaviorist prejudices” (Bever, 2020).

We know how these two researchers solved that publication problem; they founded their ideal journal, this very one you are reading now. *Cognition* the journal was explicitly aimed at stimulating theory and attracting (even bringing into being) a new generation of theorists. Note that if the whole field of science (including experimental psychology) had listened carefully enough to Charles Darwin a century earlier, it would have been impossible for journals in the 20th century to have been so obdurately empiricist, and presumably there would then have been no need for *Cognition* to be called into being.

Did the vision of the journal's founders materialise? Yes indeed. Our field is now without doubt more theory-driven than it was then. Moreover, many of those theories are now formulated as computational models. Great progress has been made and continues to be made.

But even now, one question still receives little attention: what exactly is a theory supposed to do? Here we will suggest an answer to this question, drawing examples from research on the recognition of words. Our answer will be that a theory of cognition must be an explanation, not just a description of how things work (notwithstanding that that is a good place to start!). To be a true explanation, a theory needs to explain not just how but also why things work the way they do.

## 1 Some history

In *Cognition*‘s first decade or so, the language-based theories that it published, and in particular models of the recognition of words (e.g., Marslen-Wilson & Tyler, 1980; Morton, Hammersley, & Bekerian, 1985; Norris, 1986), were verbally described and conventionally depicted by flow-chart diagrams, involving boxes connected by arrows.

The advent in the late 1980s of the PDP paradigm (parallel distributed processing, often misleadingly described as “neural” networks) raised the bar. Theories were thereafter very likely to be implemented as computer programs. In speech recognition this led for instance to the development of TRACE (McClelland & Elman, 1986), Shortlist (Norris, 1994), the revised Cohort model (Gaskell & Marslen-Wilson, 1997) and Merge (Norris, McQueen, & Cutler, 2000). The paradigm shift in fact initiated a very productive era for word recognition research (also including notable word recognition theories that were not PDP-based–e.g., the Neighborhood Activation Model [Luce & Pisoni, 1998], and Goldinger's, 1998 episodic model).

Computational models served language researchers well. They provided the inspiration for much experimental work. Importantly, as one would hope of good scientific theories in the Popperian tradition, sometimes the models made predictions that were wrong (where “wrong” means that a prediction did not align with how participants actually behaved in a given experiment). One such case recounted by Norris (2005) shows how a mismatch of this kind could then usefully drive further modelling developments and further experiments. A word-spotting experiment (“press the button when one of the nonsense strings you will hear has a real word embedded in it”) of Cutler and Norris (1988) had included the string *jumpev* [ʤʌmpɘv]. As expected, participants responded to that item by spotting the word *jump*. But when the stimuli were presented to the computational word-recognition model Shortlist, the model spotted two embedded words – *jump* and *jumper*. Of course, the model's behaviour was totally correct. The experiment was carried out in British English (a non-rhotic language), in which *jumper* is pronounced [ʤʌmpɘ] and is thus indeed embedded in *jumpev*. But the participants' behaviour was also totally correct – by definition. So how did the participants miss that undeniably present word? More alarmingly for the present authors, how had we as experimenters missed it ourselves?

The answer to that question was delivered by what we eventually termed the Possible Word Constraint (Norris, McQueen, Cutler, & Butterfield, 1997). This constraint restricts listeners from segmenting an utterance in a way that leaves a residue that is not a possible word (such as the solitary [v] that would result from parsing *jumpev* as *jumper* + v). Not postulating impossible words is a sensible strategy for lexical segmentation, and unsurprisingly it has proven (so far) to be true not only in the 1997 British English study but also in American English (Newman, Sawusch, & Wunnenberg, 2011), Dutch (McQueen & Cutler, 1998), German (Hanulikovà, McQueen, & Mitterer, 2010), French (Dumay, Frauenfelder, & Content, 2002), Sesotho (Cutler, Demuth, & McQueen, 2002), Japanese (McQueen, Otake, & Cutler, 2001), and Cantonese (Yip, 2004); moreover, by the age of 12 months infants know to deploy it (Johnson, Jusczyk, Cutler, & Norris, 2003). Utterances are composed of a series of words (even if some of those words might not yet be in the listener's vocabulary), and words are more than a single consonant, so a parse that leaves consonants unattached and unaccounted for is almost certainly wrong. This discovery arose only because we had a computational model, and because that model, built without any regard to this constraint, proved to be wrong. (Fortunately, the model was easy to fix, and even became simpler as a result.)

But “all observation must be for or against some view”, as Darwin wrote. The development of all of the models of this time focussed primarily on the question of *how* spoken word recognition operates, with less consideration for why (though PDP models were guided in some cases by a preference for models which could be described as brain-like in their operation). Asking how something works seems like an obvious place to start, but postulating the reasons *why* it works the way it does is essential for a full explanation of any cognitive process. Our interpretation of the Possible Word Constraint, for instance, justifies its existence by pointing to the fact that without it, speech segmentation could produce candidate parses containing impossible words; producing such outputs is assumed to be a Bad Thing. Answering the question of how spoken word recognition works includes asking how it *should* work.

## 2 The teleological approach: How should things work?

This question has its origins in the Rational Analysis framework developed by John Anderson (Anderson, 1990, Anderson, 1991) and in Bayesian ideas such as the concept of the ideal observer (see Geisler, 2004, Geisler, 2011). In Rational Analysis the aim is to explain behaviour in terms of the function that needs to be performed. Clearly this is analogous to earlier approaches in the cognitive science literature, for instance to the computational level of analysis in the framework proposed by Marr (1983). An ideal observer is a more formal specification of a system that performs a particular task in an optimal manner, usually subject to some constraints on the information available. Both place an emphasis on teleological explanations, i.e. on understanding how things ought to work.

A paradigmatic example of Rational Analysis is Anderson and Schooler's (1991) work on the power law of forgetting. Given that the time-course of forgetting follows a power law, one could simply accept that as a property of memory which must be built into any model. However, Anderson and Schooler analysed a real-life instance of a sequence presumably determined by forgetting, namely intervals between the recurrence of topics in the *New York Times*. Those intervals also followed a power law. Anderson and Schooler suggested that the forgetting function was adapted to optimise access to past episodes according to the probability that those memories would be needed in the future. The forgetting function is thus not arbitrary, but is adapted to the contingencies of the environment. On the assumption that the appearance of topics in the *New York Times* is a true reflection of the need to access information over time in everyday experience, this need explains *why* forgetting follows a power law.

In theory, there is an infinite number of models that could fit any given set of data, and the sets of data relevant to any cognitive function run from developmental (the trajectory of first learning) through mature performance (in the word processing case, not only recognition but production) to functional loss (which may also proceed function by function). Each type of evidence can potentially inform the others. Inevitably it can be a challenge to construct a model of any sort at all. But where should one begin the process of searching through this potentially vast space of models, and how should choices be made between candidate theories that might fit the data equally well?

The ideal observer model provides a starting point: Develop a model of how a particular task *should* be performed and ask how well that approximates the actual behaviour. This is the rationale behind accounts such as Feldman, Griffiths, and Morgan's (2009) model of the perceptual magnet effect; the Bayesian Reader model of visual word recognition (Norris, 2006) and its companion, the Shortlist B model of spoken word recognition (Norris & McQueen, 2008); Kleinschmidt and Jaeger's (2015) model of perceptual learning and adaptation; and a number of models of lexical acquisition (e.g. Goldwater, Griffiths, & Johnson, 2009; Xu & Tenenbaum, 2007). Common to all of these models is that they are Bayesian. Ideal observer models are invariably Bayesian as the ideal or optimal way to perform many tasks is by Bayesian inference.

The concept of optimality – or maybe just the word optimality – should be applied with caution. Models can be optimal only with respect to some cost-benefit function defined for the process in question (see Griffiths, Chater, Norris, & Pouget, 2012). In Shortlist B and in the Bayesian Reader, optimality is defined as recognizing words as quickly as possible given some acceptable level of accuracy. That cannot constitute a complete specification of the cost function for a real biological system; for instance, trying to recognize words too fast might turn out to have a major metabolic cost. If this were so, then the ideal observer model would be unlikely to give a good account of the data. In that sense, ideal observer models are tools as much as theories. If the model fits the data then that explains why the system works that way. If it doesn't, then that points in the direction of other factors that may be limiting performance.

A good example of this approach, and one of the most ingenious applications of the ideal observer concept, was provided by Pelli, Farell, and Moore (2003), who took this approach to resolve the long-standing debate as to whether reading can be based on whole words, or depends on first identifying individual letters. The title of their paper, “The remarkable inefficiency of word recognition”, gives a clue to their findings. People performed much worse than would be predicted by an ideal observer model that used all of the information available (the shape of the whole word). Instead, the data were better fitted by a model that assumed that reading must proceed by first identifying individual letters independently of each other. It was the failure of the putative ideal that provided the insights into how the reading process worked.

As this example attests, Bayesian or ideal observer models are not usually developed in the belief that people are in any way actually ideal or optimal. (They often get close! But it cannot be counted upon.) Rather, that assumption of optimality is purely pragmatic: it just offers a good place to start the search for an explanation of behaviour.

The move from connectionist modelling to Bayesian modelling is a path we have taken ourselves. The earlier Shortlist (Norris, 1994) and Merge (Norris et al., 2000) models were constructed with a connectionist architecture. To the extent that they indeed simulated the data, we might consider them to have been successful. But they had lots of parameters and various assumptions that in essence existed just for one reason: to make them work. Like most connectionist models, they focussed on the how rather than the why. In contrast, Shortlist B (Norris & McQueen, 2008) is Bayesian. It does everything the other models can do, and more, in a simpler model with fewer parameters. Carefully chosen parameters determining activation and inhibition disappeared, to be replaced entirely by Bayes' theorem. Note that there is no incompatibility between Bayesian theorisation and connectionist implementation. Connectionist networks can implement Bayesian computations (e.g. McClelland, 2013). Thus it would be possible to build a connectionist model that computed exactly the same functions as Shortlist B, but the parameters in the connectionist model would be fixed by the need for the model to perform strictly in accord with Bayesian guidelines. Why not use those guidelines directly rather than indirectly? Operations that required 22 parameters in the TRACE model were equally successfully simulated with just two parameters in Shortlist B.

We conclude by emphasising that we are advocating a teleological approach as a strategy for the development of theories and models. It is not a theoretical claim that all behaviour has evolved to be ideal (see for example Gould and Lewontin's (1979) critique of the adaptationist view in evolutionary theory). As the example from Pelli et al. illustrates, the ideal observer approach can provide important theoretical insights even when human behaviour is manifestly sub-optimal.

## 3 Payoff abounding (or: for instance, why feedback?)

The teleological framework necessarily encourages parsimony. An optimal solution cannot include unnecessary processes that exist simply because the theorist thinks they are a good idea. This has been pointed out for models of various cognitive functions, with a particularly strong case, for instance, being made for visual perception by Firestone and Scholl (2016). In the case of speech perception, the most frequently encountered example is the claim that recognition benefits from interaction between different levels of processing, including feedback of activation from lexical to pre-lexical processing (“activation feedback”).

Such interaction in fact serves no useful function at all and cannot improve recognition outcomes. Consider how feedback from lexical to phonemic processing is embodied in the TRACE model (McClelland & Elman, 1986); when a word is activated, that word sends activation back to the earlier stage (phoneme recognition) and activates the word's constituent phonemes. In turn, these phonemes send yet more activation on to the lexical level. But what does this achieve? Activation from the (already best matching) lexical items has flowed back down to the phonemic representations and further increased their activation, and this can then further activate the same lexical items. Since these were already the best matching candidates, recognition does not improve; all that happens is reinforcement of the status quo. So why is such feedback, that produces no advantage, included in the model? A recognition system should identify the word that best matches the input, and this has already happened, i.e., an interactive activation system is quite capable of performing this matching process on the basis of the feedforward connections alone.

It is sometimes argued that activation feedback is required to ensure that recognition processes are more robust should speech be degraded. So, can recognition of a degraded signal be improved by the kind of activation feedback incorporated in TRACE? The answer is again no. The above argument applies regardless of whether the input is degraded. The degraded-speech case still regularly causes confusion (e.g., Magnuson, Mirman, Luthra, Strauss, & Harris, 2018), and may have its origin in the original version of TRACE, which ruled out noise both in the input and during processing. (This was never intended as a theoretical claim about how speech recognition works; it just helped to simplify the model for computational purposes. The effect of noise was simulated by adding a constant amount of noise to a decision process – the Luce choice rule – operating on the output of the network.) In such a system, where feedback and noise are operating at different levels, feedback (as part of processing) can alter the relative activation of a word and its competitors but at the same time have no effect at all on any noise (which operates separately on final outputs). In other words, because of a workaround in the model, simulations using TRACE can give the impression that feedback can improve performance. This could never be true of a real biological system, however. In a real system with degraded input, signal and noise are in the same processed channel, and feeding activation back to the phoneme level will boost both the signal and the noise equally. Feedback can here do nothing to improve the signal to noise ratio. Note that this argument was laid out and fully agreed decades ago in a debate between Massaro (1989) and McClelland (1991; see also 2013).Activation feedback is not the only way in which later-level processing outcomes can inform earlier processing level operations, however. Communication of later processing results can be vitally useful, most obviously for learning. Consider that listeners often encounter speakers with an unfamiliar regional accent, or with an articulatory system that leads to certain segments being pronounced in an unusual manner. Suppose a speaker produces an unknown name containing a sound that is ambiguous between [s] and [f]: “Have you met *Mr. Re*[f/s]*ton?”* Listeners will have no way of knowing which phoneme is intended: is this *Mr. Refton* or *Mr. Reston*? However, if the speaker produces the same ambiguous sound in *hor*[f/s] or *gira*[f/s], then an English-speaking listener can rely on accrued experience with the words of English and be fairly sure that the intended phoneme was [s] (in the case of *horse*) or [f] (in the case of *giraffe*). In principle, an ideal listener would be able to use that information to retune the category boundaries applying for that speaker. That is, lexical knowledge can be drawn upon to tailor sub-lexical processing of future utterances from the same speaker.

In line with this expectation, Norris, McQueen, and Cutler (2003) showed that this is exactly what happens. Listeners use this strategy, which effectively involves communication from later to earlier processing levels, because word recognition is improved by using it. Note also that this learning is itself evidence against activation feedback; if activation from the lexical level were to control the outcome of processing at the pre-lexical level, there would be no need for adjustment of the pre-lexical category decisions, because no difference in processing as a function of speaker identity would ever be registered. The learning would never be needed if activation feedback could auto-correct decisions in every such situation of ambiguity. But the learning does occur; listeners do indeed adjust the phonetic category boundaries to improve future listening to individual speakers.

There is a major difference between these two kinds of higher-to-lower transfer of information. Activation feedback was assumed to operate during the process of recognizing each word; but such activation just re-asserts the status quo. Learning, in contrast, happens after the word triggering it has been recognised. The listener learns from the perceptual experience, and retunes perception, whereby word recognition is improved in the future. If any and every higher-to-lower transfer of information is to be categorised as feedback, then this feedback, for learning, is feedback with a rationale, and as such, it should obviously have a place in any model.

## 4 Where now (a summary, but also with some retrospection)

We began by asking what a theory should do. We suggested that theories should constitute an account of how things work, but accompanied, crucially, by an explanation of *why* the proposed account has the properties it does. This procedure is not just a search for a deeper level of understanding; the teleological approach is a way of guiding the scientific process itself.

Thus consider starting out with the goal of developing a theory of (oh, let's take an example at random!) word recognition. The space of possible theories is vast. One might start by making a guess about the kind of mechanism involved and how to test it, or perhaps choose for external reasons to build a particular type of architecture such as a neural net. But beyond that lie still an infinity of details that need to be decided, and where does one even begin? The teleological approach provides a pathway through that theory space, because it suggests where to start: by assuming that people behave rationally, or as ideal observers. That is still far from the end, of course. If people really behaved ideally in all respects, cognitive psychologists would be out of business. (Once we knew how a task should be performed, there'd be no need to look any further.) So the budding theorist must next look for ways in which word recognition behaviour departs from the ideal. As the example provided by Pelli et al. (2003) shows, it is departures from the ideal that most compellingly provide insights into how cognition really works. *Should* is defined by *why* and *should* can tell us *how*; a theory is the vehicle that brings this all together.

Our own work with Jacques Mehler (and Juan Seguí) in the 1980s represented an attempt to follow this path. Our first joint project addressed a major issue of that faraway day: what were the “units of perception”? That is, how did the listener turn an incoming continuous speech stream into a form which would enable access to the words stored in the mental lexicon? What we found was unexpected both by us and by the reigning preconceptions; only after several studies (Cutler, Mehler, Norris, & Seguí, 1983; Cutler, Mehler, Norris, & Seguí, 1986; Cutler, Mehler, Norris, & Seguí, 1989; Cutler, Mehler, Norris, & Seguí, 1992) could we accept that there was one answer for listeners with French as their native language, but another answer for those whose native language was English. The “how” in our model thus had more than one answer, and nothing was more sure than that this cried out for a “why” to explain it! In the later of those publications we suggested that a unifying factor could be the rhythmic structure of each language (rhythm being the crucial signature of language identity for listeners from their earliest listening experience, even before birth; Cutler & Mehler, 1993). Indeed later research (blessed with the support of the then newly founded Human Frontier Science Foundation) led to us bolstering this suggestion with evidence from segmentation in Japanese, a language with a rhythmic structure unlike either that of French or that of English (Otake, Hatano, Cutler, & Mehler, 1993).

Our discussion of the role of feedback has provided evidence that, despite the best attempts of many, the importance of theory – the *why* that justifies our models – still isn't always fully appreciated in cognitive psychology. In his account of the foundation of this journal (Mehler, 1994), Jacques remarked drily that the unspoken guideline of most journals prior to the cognitive revolution was “Make your introduction as short and vacuous as possible”. The word limits in many high-profile journals today ominously suggest that such a goal is often still held in esteem. But since the mid-1970s, research on cognition has had at least one space where theory is central, and welcomed, and celebrated. Thank goodness for *Cognition*, and thank goodness for Jacques, who steadfastly encouraged us to fill that theoretical vacuum.
